# Noninvasive diagnosis of AIH/PBC overlap syndrome based on prediction models

**DOI:** 10.1515/med-2022-0526

**Published:** 2022-09-28

**Authors:** Kailing Wang, Yong Li, Jianfeng Pan, Huifang He, Ziyi Zhao, Yiming Guo, Xiaomei Zhang

**Affiliations:** Department of Gastroenterology, Xiangya Hospital, Central South University, Changsha, Hunan 410008, China; National Clinical Research Center for Geriatric Disorders, Xiangya Hospital, Central South University, Changsha, Hunan 410007, China

**Keywords:** primary biliary cirrhosis, predictive model, immunoglobulin

## Abstract

Autoimmune liver diseases (AILDs) are life-threatening chronic liver diseases, mainly including autoimmune hepatitis (AIH), primary biliary cholangitis (PBC), and AIH–PBC overlap syndrome (OS), which are difficult to distinguish clinically at early stages. This study aimed to establish model to achieve the purpose of the diagnosis of AIH/PBC OS in a noninvasive way. A total of 201 AILDs patients were included in this retrospective study who underwent liver biopsy during January 2011 to December 2020. Serological factors significantly associated with OS were determined by the univariate analysis. Two multivariate models based on these factors were constructed to predict the diagnosis of AIH/PBC OS using logistic regression and random forest analysis. The results showed that immunoglobulins G and M had significant importance in both models. In logistic regression model, anti-Sp100, anti-Ro-52, anti-SSA, or antinuclear antibody positivity were risk factors for OS. In random forest model, activated partial thromboplastin time and ɑ-fetoprotein level were important. To distinguish PBC and OS, the sensitivity and specificity of logistic regression model were 0.889 and 0.727, respectively, and the sensitivity and specificity of random forest model were 0.944 and 0.818, respectively. In conclusion, we established two predictive models for the diagnosis of AIH/PBC OS in a noninvasive method and they showed better performance than Paris criteria for the definition of AIH/PBC OS.

## 1 Introduction

Autoimmune hepatitis (AIH), primary biliary cholangitis (PBC), and primary sclerosing cholangitis are main categories of autoimmune liver diseases (AILDs) caused by anomalous response of immune system to self-antigens on hepatocytes or bile ducts. AIH and PBC have different mechanisms and clinical manifestations. In AIH, autoimmune injury mainly affects the hepatocytes, leading to the presence of interface hepatitis in liver histology, and AIH is characterized by high levels of immunoglobulin G (IgG) or γ-globulin and some positive serum autoantibodies [1]. PBC is characterized by circulating anti-mitochondrial antibodies (AMAs), chronic cholestasis, and autoimmunity on the intrahepatic small bile ducts, and the liver biopsy findings show typical appearance of non-purulent granulomatous destructive cholangitis [2,3]. About 2–19% of patients share overlapping features of both PBC and AIH, and it is called PBC–AIH overlap syndrome (OS) [4,5,6,7]. OS may represent an important and unrecognized cause of resistance to ursodeoxycholic acid in patients with PBC [6]. If treated inappropriate, OS leads to the development of liver cirrhosis rapidly and even liver failure, which need liver transplantation [8]. Therefore, early diagnosis and proper treatment of OS are extremely important.

The sensitivity and specificity of the Paris criteria for OS were reported to be 92 and 97%, respectively [9]. However, patients with less severe forms of AIH–PBC OS may not be captured by the Paris criteria [10]. The gold standard for diagnosing AILDs is still liver pathology, which can evaluate the severity and prognosis and determine treatment options [11,12]. However, liver biopsy has some disadvantages. First, about one quarter of patients already develop cirrhosis at diagnosis [13], have poor blood clotting and a greater risk, and increase the possibility of complications. Second, sampling process errors occur in needle liver biopsies of AILDs, which may be affected by different extents of lesions. Finally, OS should not be over-diagnosed to avoid the risk of steroid side effects on PBC patients [14,15].

Therefore, a noninvasive method as a supplementation for biopsy is urgently needed to differentiate PBC and OS, which should be safe, convenient, accurate, and effective. This noninvasive approach helps integrate numerous clinical parameters, providing a reliable understanding of liver pathology features and prognosis. The artificial intelligence model is well suited to this challenge and has already been used to predict effects of different treatment options [16,17,18,19].

This study aimed to develop and validate a prediction model for predicting the diagnosis of AIH/PBC OS based on machine learning models. We utilized the AILD patient cohort and selected valuable parameters and weighed for their importance.

## 2 Patients and methods

### 2.1 Patient and public involvement

It was not appropriate or possible to involve patients or the public in the design, or conduct, or reporting, or dissemination plans of our research.

This study was approved by Ethics Committee of Xiangya Hospital of Central South University (Changsha, China, Approval ID: 20341), and all patients provided informed consent. A total of 201 patients with AILDs admitted to Xiangya Hospital between 2011 and 2020 were retrospectively analyzed.

Patients with AIH were selected based on the international group for the study of AIH simplified criteria [20]. These simplified diagnostic criteria for AIH have been validated in different countries including China [21,22,23]. PBC patients were classified according to the Paris criteria [10]. PBC–AIH OS was strictly defined by the association of PBC and AIH either simultaneously or consecutively [9]. In each patient, the absence of biliary obstruction was assessed by ultrasonography and hepatitis virus serology, and copper blue protein was negative. None had excessive alcohol consumption (<20 g/day), and there was no evidence of exposure to hepato- or bile duct toxicity. To ensure an accurate classification of AILDs, all patients had pathological results of liver biopsy. Among 201 patients with AILDs, 65 patients were excluded according to the exclusion criteria as follows: age <18 years (*n* = 2), virus hepatitis (*n* = 52), drug-induced liver injury (*n* = 3), liver cancer (*n* = 2), blood disease (*n* = 1), and incomplete information (*n* = 5).

### 2.2 Collection of clinical and pathology data

Information on each patient, including history as well as symptoms, clinical findings, and data from laboratory or other diagnostic investigations were obtained, including age and sex; clinical symptoms including pruritus, jaundice, and fatigue; red blood cell (RBC), white blood cell (WBC), platelet (PLT), lymphocyte count (L), eosinophil count (E), basophil count (B), monocyte count (M), aspartate aminotransferase (AST), alkaline phosphatase (ALP), γ-glutamyl transferase, international normalized ratio (INR), activated partial thromboplastin time (APTT), IgG, immunoglobulin A (IgA), immunoglobulin M (IgM), and auto-antibody tests. All auto-antibody tests were performed using Western blot-based antibody detection kits (Oumeng Diagnostics Ltd., Germany).

### 2.3 Statistical analysis

Continuous variables were described as median and interquartile range (IQR), and categorical variables were described as absolute frequencies and percentages. SPSS 25.0 was utilized for statistical analysis. The chi-squared test or Fischer’s exact test was used to compare categorical data. For the parametric distribution, Student’s *t*-test was used to compare the mean values of two groups. For nonparametric variables, the Kruskal–Wallis test or Mann–Whitney *U* test was used. All *P*-values were two-sided, and *P*-values <0.05 were considered statistically significant.

### 2.4 Model establishment

A random forest model was constructed to distinguish PBC from OS using the statistical language R (version 3.3), with the packages random forest, pROC, and rms. The random forest creates multiple training sets for decision trees, wherein each tree is built based on a bootstrap sample drawn randomly from the original dataset using the classification and regression tree method and the decrease Gini impurity as the splitting criterion [24]. Of the final cohort included, 70% were selected for training and the remaining for validation. A 10-fold cross-validation strategy was performed during training. In the model, we determined 500 decision trees in the forest, and 5 variables were considered on each decision tree. The maximum depth of the trees was set to 3 [25]. The confidence intervals for area under the receiver-operating characteristic curve (AUC) and other evaluation criteria were constructed using bootstrapping. The one that produced an AUC closest to the average was considered the best trained model. The model was then verified in the testing dataset. We selected the AUC, accuracy, sensitivity, and specificity to evaluate the performance of the model. The input variables in our model were ranked by relative importance in diagnosing PBC based on the mean decrease in accuracy and the mean decrease in the Gini coefficient [26]. Moreover, we developed the logistic regression model using the same training data and testing data.

## 3 Results

### 3.1 Demographics and baseline features

**Figure 1:**
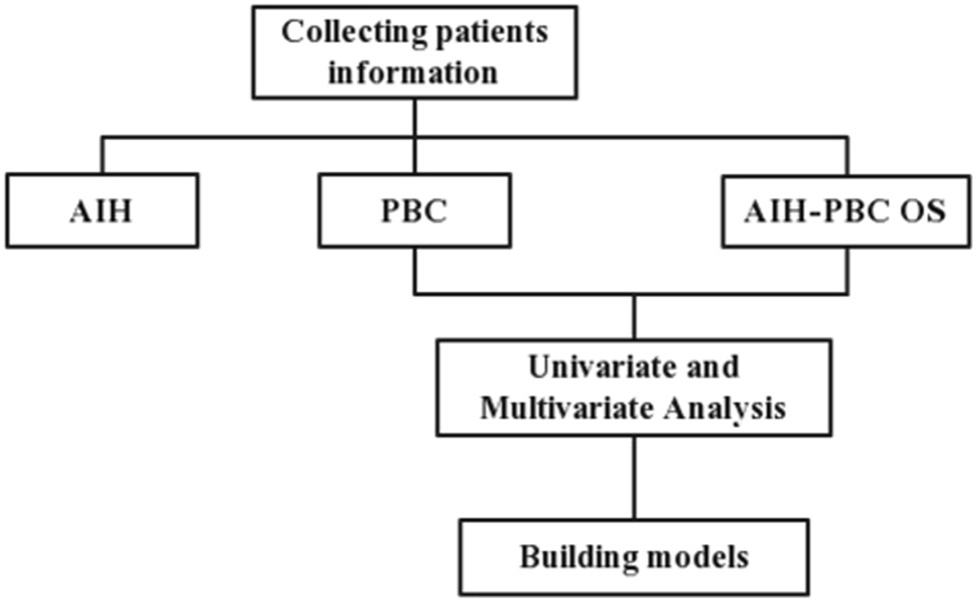
Brief scheme of the study design.

The medical records of 201 AILD patients with liver biopsy were reviewed. Finally, 136 patients were enrolled in this study and divided into the PBC group (35, 25.74%), the AIH group (44, 32.35%), and the OS group (57, 41.91%) (Figure 1). We found no difference in the distribution of age or sex among the three groups. The general characteristics of the study participants are summarized in Table 1.

**Table 1:** Demographic characteristics of study subjects

Variables	AIH	(*N* = 44)	PBC	(*N* = 35)	OS	(*N* = 57)	P*-value
Female	41	(93.2%)	27	(77.1%)	48	(84.2%)	0.129
Age, years	51.3	(10.1)	47.5	(12.0)	50.1	(9.6)	0.504
**Symptoms**							
Jaundice	23	(52.3%)	22	(62.9%)	37	(64.9%)	0.406
Pruritus	0	(0%)	5	(14.3%)	7	(12.3%)	0.04
Fatigue	12	(27.3%)	7	(20%)	17	(29.8%)	0.584
**Signs**							
Liver palms	5	(11.4%)	1	(2.9%)	9	(15.8%)	0.154
Spider angioma	4	(9.8%)	1	(2.9%)	7	(12.3%)	0.273
Lower limb edema	5	(11.4%)	6	(14.6%)	4	(7.0%)	0.325
Imaging results							
Hepatomegaly	5	(11.4%)	6	(14.6%)	13	(22.8%)	0.319
Splenomegaly	20	(48.8%)	17	(48.6%)	45	(79.0%)	0.001
Ascites	10	(24.4%)	13	(31.7%)	12	(21.1%)	0.218
**Complications**							
Other AIDs	8	(18.2%)	8	(22.9%)	14	(24.6%)	0.753
Gallbladder diseases	22	(50.0%)	14	(40.0%)	21	(36.8%)	0.412
Liver cirrhosis	17	(38.6%)	12	(34.3%)	33	(57.9%)	0.045
Varicose veins	4	(9.1%)	4	(11.4%)	15	(26.3%)	0.046
Infection	7	(15.9%)	6	(17.1%)	10	(17.5%)	0.976

Data are median (IQR) or *n* (%). A *P*-value of less than 0.05 is considered statistically significant among three groups.

*The *P*-value represents comparison between AIH, PBC, and OS within the test panel.

### 3.2 Comparison between AIH and other liver diseases

To explore the difference between AIH and other ALDs, we performed single-factor and multi-factor comparisons. The blood indexes of study subjects are shown in Table 2. In immunological index, more AIHs were positive for antibodies against M2-3E (BPO), while more PBC and OS were positive for AMA, and antinuclear antibody (ANA) showed significant differences. Sjogren’s syndrome (SS)-related SSA was associated with PBC [odds ratio (OR) 5.5, *P* = 0.02]. RO-52 was associated with OS (OR 0.26, *P* = 0.002).

**Table 2:** Blood indexes of patients studied

Parameters	AIH	(*N* = 44)	PBC	(*N* = 35)	OS	(*N* = 57)	P*-value
WBC (109/L)	4.65	(1.67)	5.1	(2.3)	4.4	(2.4)	0.621
RBC (1,012/L)	4.07	(0.82)	3.9	(1.13)	3.7	(0.6)	0.114
Hb (g/L)	122.5	(33.5)	115.0	(32.0)	113	(28.5)	0.522
PLT (109/L)	161	(118.75)	166	(108.0)	166	(105.5)	0.727
N (109/L)	2.7	(1.13)	3	(2)	2.7	(1.5)	0.392
L (109/L)	1.3	(0.8)	2.3	(1.6)	1.78	(1.2)	<0.001
E (109/L)	0.1	(1.18)	1.4	(0.8)	1.5	(0.7)	<0.001
B (109/L)	0	(0)	0.1	(0.1)	0.1	(0.19)	<0.001
M (109/L)	0.55	(0.3)	0.07	(0.10)	0.08	(0.11)	<0.001
NLR (%)	178.63	(95.98)	231.82	(150)	178.26	(116.57)	0.337
ELR (%)	5	(11.11)	6.25	(10)	8	(10.55)	0.353
BLR (%)	0	(0)	0	(0)	0	(1.52)	0.42
MLR (%)	39.23	(27.74)	27.78	(21.43)	27.27	(0.213)	0.008
LPR (%)	0.88	(0.54)	0.82	(0.58)	0.91	(0.46)	0.451
Albumin (g/L)	35.5	(9.83)	38.2	(9.3)	38.5	(7.65)	0.066
Globulin (g/L)	38.65	(10.68)	34.6	(10.3)	43.8	(8.35)	<0.001
TBIL (mol/L)	40.3	(84.68)	31.9	(54.6)	39.2	(50.1)	0.357
DBIL (mol/L)	24.25	(46.88)	19.8	(36.1)	20.4	(30)	0.467
Bile acid (mol/L)	37.6	(129.43)	52	(109.8)	41.5	(59.05)	0.989
ALT (U/L)	127.85	(322.55)	87.9	(121.8)	95.3	(96.65)	0.078
AST (U/L)	159.15	(327.58)	96	(95.99)	133.5	(102.75)	0.042
AAR (%)	129.74	(82.75)	120.19	(88.1)	139.41	(71.73)	0.487
ALP (U/L)	164.75	(167.85)	443	(430.3)	367.4	(518.3)	<0.001
γ-GT (U/L)	151.8	(141.29)	381	(592.4)	348.8	(425.1)	<0.001
5-Nucleotidase (U/L)	10.75	(14.65)	36.9	(95.7)	51.9	(69.1)	<0.001
Fucosidase (U/L)	37.25	(16.82)	42.5	(25.8)	41.7	(27.35)	0.532
mAST (IU/L)	29.55	(40.64)	27.5	(30.4)	34.8	(24.8)	0.375
Triglicerides (mmol/L)	1.54	(0.82)	2.1	(0.98)	1.7	(0.89)	0.02
Cholesterol (mmol/L)	4.16	(1.48)	6.4	(3.84)	6.07	(2.39)	<0.001
HDL (mmol/L)	1.005	(0.66)	1.56	(0.97)	1.63	(0.9)	<0.001
LDL (mmol/L)	2.38	(0.86)	4.1	(2.1)	3.51	(1.46)	<0.001
PT (s)	14.4	(3.25)	12.7	(1.6)	13.3	(2.25)	<0.001
INR	1.13	(0.27)	0.99	(0.14)	1.05	(0.16)	<0.001
APTT (s)	40.27	(7.1)	33.9	(9.0)	38.16	(7.8)	0.002
Fibrinogen (g/L)	2.22	(0.92)	3.15	(1.27)	2.9	(1.01)	<0.001
D-dimer (mg/L)	0.25	(0.15)	0.16	(0.22)	0.18	(0.16)	0.238
K (mmol/L)	3.72	(0.7)	3.86	(0.42)	3.74	(0.41)	0.217
Na (mmol/L)	139.85	(3.05)	141.55	(3)	140.3	(4.6)	0.221
Cl (mmol/L)	104.17	(3.08)	104.27	(5)	103.7	(4.8)	0.621
Ca (mmol/L)	2.22	(0.15)	2.29	(0.2)	2.23	(0.16)	0.027
P (mmol/L)	1.12	(0.24)	1.17	(0.25)	1.12	(0.21)	0.373
Mg (mmol/L)	0.84	(0.09)	0.84	(0.11)	0.84	(0.08)	0.881
C3 (mg/L)	152	(75.5)	194	(118)	192	(79)	0.012
C4 (mg/L)	846.5	(346.5)	1,130	(564)	1,030	(335.5)	<0.001
IgG (g/L)	22.25	(10.55)	14.3	(4.9)	22.4	(6.4)	<0.001
IgA (mg/L)	3,220	(2,265)	2695.52	(1,840)	3740	(2,060)	0.002
IgM (mg/L)	1,570	(1102.5)	2,430	(2,220)	3,950	(2,635)	<0.001

Data are median (IQR). Hb, hemoglobin count; N, neutrophil count; NLR, neutrophil lymphocyte ratio; ELR, eosinophil lymphocyte ratio; BLR, basophil lymphocyte ratio; MLR, monocyte lymphocyte ratio; LPR, lymphocyte platelet ratio; TBIL, total bilirubin; DBIL, direct bilirubin; AAR, AST and ALT ratio; γ-GT, γ-glutamyl transferase; mAST, mitochondrial aspartate aminotransferase; C4, complement C4; C3, complement C3.

*A *P*-value of less than 0.05 is considered statistically significant.

We put the variables with *P*-values less than 0.05 in the univariate analysis into the logistic regression analysis. The AUC between AIH and PBC and between AIH and OS was 1.

Comparison between PBC and OS showed that Sp100, RO-52, and SSA were associated with OS: Sp100 (OR [95% confidence interval (CI)]: 0.30 [0.09–0.99], *P* = 0.041), RO-52 (OR [95% CI]: 0.313 [0.13–0.77], *P* = 0.01), and SSA (OR [95% CI]: 0.12 [0.03–0.56], *P* = 0.002) (Table 3).

**Table 3:** Comparison between PBC and OS

Parameters	PBC	(*N* = 35)	OS	(*N* = 57)	*P*-value
Sp100 (*n*, %)	4	(11.43%)	17	(29.82%)	0.041
RO-52 (*n*, %)	10	(28.57%)	32	(56.14%)	0.010
SSA (*n*, %)	2	(5.71%)	19	(33.33%)	0.002
ANA (*n*, %)	7	(20.00%)	0	(0.00%)	0.006
Globulin (g/L)	34.6	(10.3)	43.8	(8.35)	0.000
IgG (g/L)	14.3	(4.9)	22.4	(6.4)	0.000
IgA (mg/L)	2695.52	(1,840)	3,740	(2,060)	0.001
IgM (mg/L)	2,430	(2,220)	3,950	(2,635)	0.002

A *P*-value of less than 0.05 is considered statistically significant.

### 3.3 Construction of model to discriminate PBC and OS

**Figure 2:**
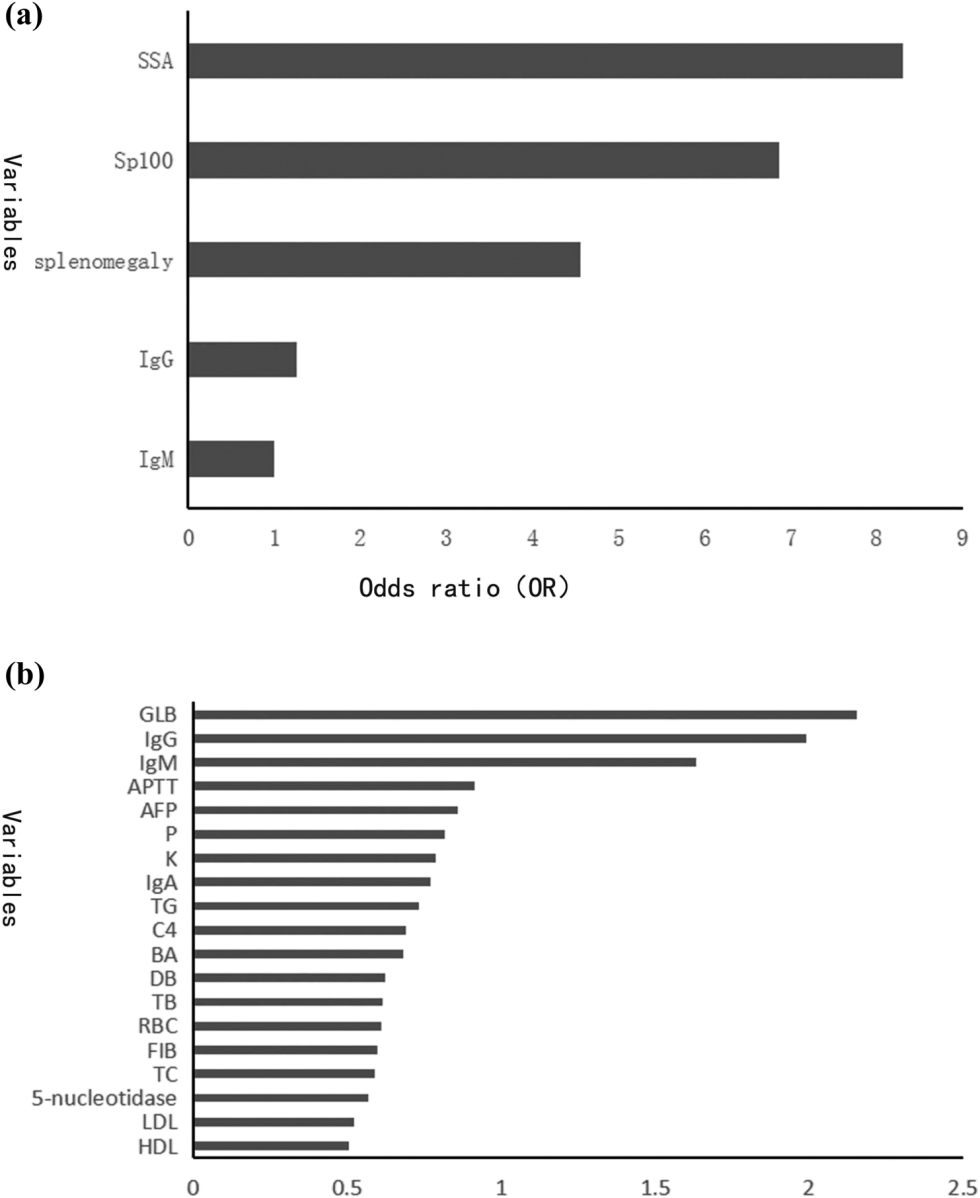
The importance ranking of variables predicting the diagnosis of PBC and OS. (a) OS-related variables ranked by logistic regression analysis (*P*＜0.05). (b) OS-related variables ranked by random forest analysis. SSA, anti-SSA antibody; Sp100, anti-Sp100 antibody; P, serum phosphorus level; K, serum potassium level; TG, triglyceride; C4, complement 4; BA, serum bile acid; DB, direct bilirubin; TB, total bilirubin; FIB, fibrin; TC, total cholesterol; LDL, low-density lipoprotein; HDL, high-density lipoprotein.

**Figure 3:**
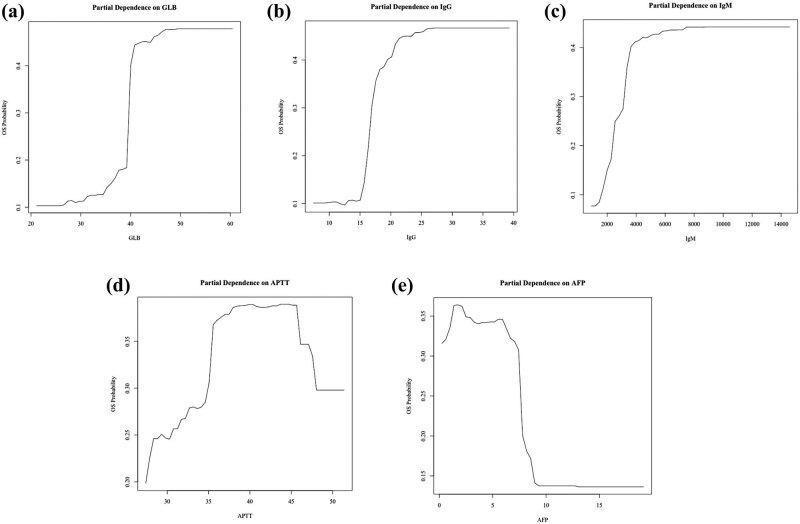
The effects of five important variables on the outcome of diagnosing OS. (a) GLB. (b) IgG. (c) IgM. (d) APTT. (e) AFP.

To construct prediction model to discriminate PBC and OS, first we ranked the variables used to construct the model in the order of importance (Figure 2a). For the model based on random forest after over 50 replications, we chose a model which displayed the receiver-operating characteristic (ROC) under the selected training and testing cohort with the AUC of 0.93 (95% CI: 0.91–0.99), the closest to the average. According to the mean decrease in the Gini value, we found that the five strongest predictor variables used to differentiate PBC from OS were ɑ-fetoprotein (AFP), APTT, globulin (GLB), IgG, and IgM (Figure 2b). After validation in the remaining 30% of samples, the random forest model could predict the outcome with the sensitivity of 0.944 and the specificity of 0.818. The effects of these five variables on the outcome of diagnosing PBC are shown in Figure 3.

**Figure 4:**
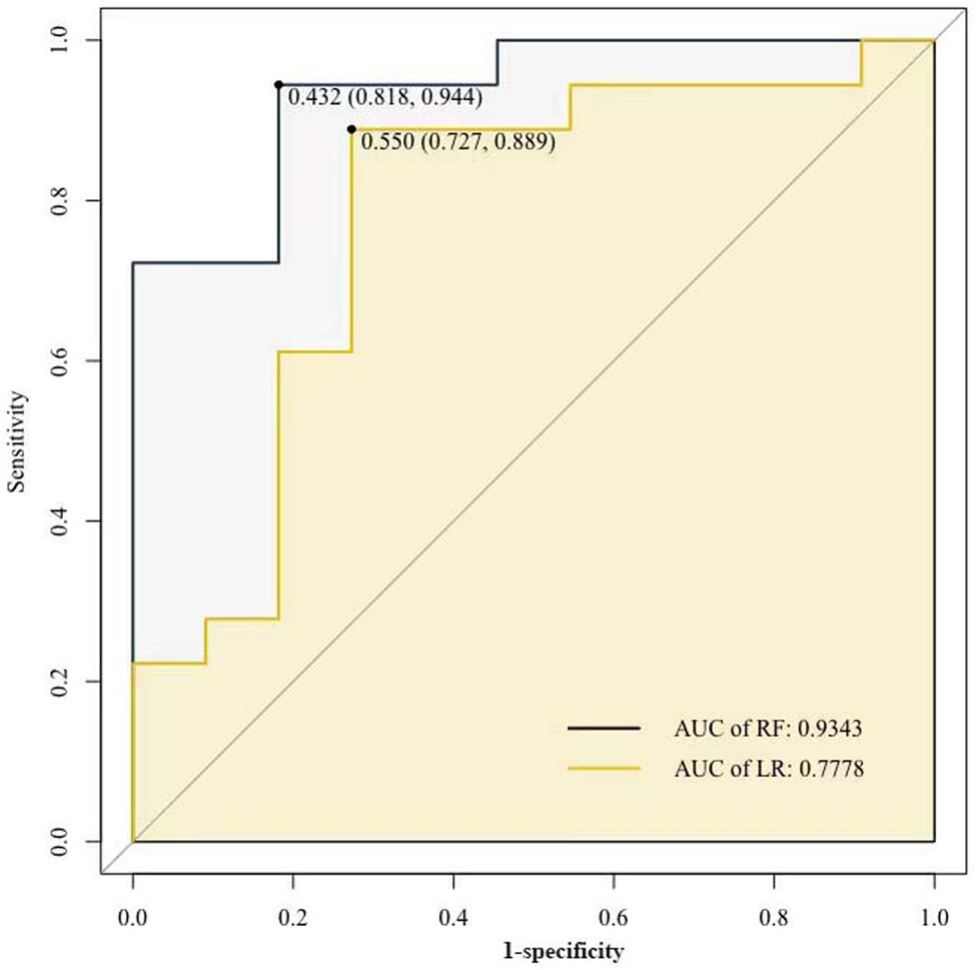
The ROC curve of predictive models for distinguish PBC with OS. RF, random forest; LR, logistic regression.

We also established prediction model applying binary logistic regression algorithms with the sensitivity of 0.889 and the specificity of 0.727 after testing in the same data. The AUC for this model was 0.78 (95% CI: 0.63–0.86), which was lower than that of the random forest model (Figure 4).

## 4 Discussion

In this retrospective study, we conducted single-factor and multi-factor analysis of patients with AILDs. When AIH was compared with PBC and OS, many variables showed significant differences, indicating that it is easy to distinguish them. However, it is difficult to distinguish PBC from OS, because the two diseases lack unique clinical characteristics [6]. In fact, there is no international consensus on the definition of OS in daily practice, especially on the histological level. The percentage of OS patients was higher than usual, which may be related to the fact that liver biopsy is not necessary for the diagnosis of PBC alone [11]. Moreover, we excluded some patients with comorbidities, which may have some selection bias. For AIH and OS, liver biopsy is necessary, and the early detection rate of OS patients has increased because of liver biopsy [12]. Therefore, we established two prediction models to reduce unnecessary operations and make effective early diagnosis. The results showed that the models had powerful competence to differentiate PBC and OS.

In our study, it is important to note that the magnitude of elevated IgG in OS was more than that in PBC. From a clinical point of view, hypergammaglobulinemia is one of the prominent clinical characteristics of AIH patients, and the decrease in IgG is an important aspect for disease control [27]. The presence of a combination of anti-double-stranded DNA (anti-dsDNA), elevated alanine aminotransferase (ALT), and elevated IgG levels should prompt a clinician to a potential diagnosis of OS [28]. We found similar result that OS is more prone to higher levels of immunoglobulins, IgG and IgM, in random forest model. Many autoantibodies have been detected in AILDs, and AMA has been recognized as specific targets of PBC [29]. However, in our study, we found no difference in AMA between PBC and OS, which may be related to the fact that OS has clinical traits of PBC. AMAs are not associated with disease progression, while ANAs are related to disease severity and clinical outcome, and are the markers of poor prognosis. In particular, ANAs are detected in up to 50% PBC patients. Two immunofluorescence patterns are considered PBC specific: the multiple nuclear dot patterns for antigens, such as Sp100 and promyelocytic leukemia protein, and rim-like/membranous patterns for antigens, such as gp210, nucleoporin p62, and the lamin B receptor [30,31]. In our study, the prevalence of ANA was 100% in AIH and 80% in PBC patients.

It was reported that sp100 had a sensitivity of 40% and a specificity of 97.3% for PBC [32]. OS also has the characteristics of PBC, and Sp100 and SSA may be potential autoantibodies to differentiate patients between PBC and OS. In fact, SSA and anticentromere antibody (ACA) are helpful for the diagnosis of PBC with SS, and SSA and ACA are recognized as serological markers of AMA negative PBC patients [33]. In our study, OS patients were more likely to have SS. Anti-dsDNA and anti-p53 have been suggested to be potential autoantibodies for identifying patients with OS [27,34].

Furthermore, gp210 antibodies in PBC are associated with severe prognosis [35]. One study found significantly higher frequency of anti-gp210 in patients with OS than in patients with PBC [36], indicating that OS has a worse prognosis. However, we did not detect significantly higher frequencies of gp210, dsDNA, and p53 in OS patients compared to PBC patients.

Logistic regression model showed that the combination of splenomegaly with Sp100, SSA, and IgG levels was able to differentiate patients with OS from those with PBC. In random forest model, APTT and AFP level were factors in the top 5. It has been reported that prothrombin time (PT) and APTT can be used as appropriate predictors of bleeding risk due to impaired liver synthesis and reduced procoagulant factors in cirrhosis [37]. However, other study showed that APTT abnormalities were poorly associated with bleeding [38]. Therefore, the importance of APTT is controversial and requires follow-up verification. Generally, the sensitivity and specificity of random forest model were better than those of logistic regression model, which is likely due to the strong generalization power of the random forest model [39].

Recently, Wang et al. developed a nice model based on limited sociodemographic and clinical parameters from routine health checkup to identify individuals at high risk for AILD [40]. In contrast, our model has unique advantages: it is noninvasive; it integrates multiple irrelevant variables simultaneously and evaluates the weight of each variable; it can be refined continuously as database enlarges and sensitive variables are constantly discovered. More importantly, our model could be used to distinguish PBC and OS. Similarly, Zhang et al. developed a scoring classification based on selected histologic features of AIH and PBC and modified biochemical and immunologic characteristics, and it showed a high sensitivity and specificity for the diagnosis of OS and may be better than current OS scoring systems to detect mild forms of OS [41].

It should be noted that our study has some imitations. First, we did not compare healthy control cohort. Second, our sample size is relatively small. Third, to improve the sensitivity and specificity of our model, we focused on the factors with importance ranking shown in Figure 1 and did not include other significant biomarkers or demographic factors, such as those used in previous study [40]. Further studies are needed to explore novel and sensitive parameters. With the enlargement of data, machine learning will gain power and become a promising approach to distinguishing PBC and OS.

In conclusion, we constructed two models with sufficient accuracy to predict the diagnosis of PBC and OS patients who probably benefit from early treatment based on readily available parameters. Following the use in clinical practice, these models help patients with early and effective treatment and reduce surveillance liver biopsies in the future.
